# Severe Sepsis With Septic Shock as a Consequence of a Severe Community-Acquired Pneumonia Resulting From a Combined Legionella pneumophila and Streptococcus pneumoniae Infection

**DOI:** 10.7759/cureus.10966

**Published:** 2020-10-15

**Authors:** Jose Orsini, Brendan J Frawley, Hannah Gawlak, Rebecca Gooch, Javier Escovar

**Affiliations:** 1 Department of Medicine, Division of Critical Care Medicine, Jacobs School of Medicine and Biomedical Sciences at University of Buffalo, Mercy Hospital of Buffalo, Buffalo, USA; 2 Department of Medicine, The Brooklyn Hospital Center, Brooklyn, USA

**Keywords:** legionella pneumophila, streptococcus pneumoniae, acute respiratory failure, acute kidney injury, rhabdomyolysis, pancreatitis

## Abstract

Community-acquired pneumonia (CAP) is a frequent cause of intensive care unit (ICU) admission in adults and the sixth leading cause of death worldwide. Although co-infections have been previously reported, the co-existence of *Streptococcus pneumoniae* and *Legionella pneumophila* is exceedingly rare. Despite the fact that *Streptococcus pneumoniae* is the most common etiology in the majority of cases, atypical organisms such as *Legionella pneumophila* should be considered as etiologic agents among all CAP cases that require hospitalization. Unlike *Legionella*, extra-pulmonary findings are uncommon in patients with *Streptococcus pneumoniae* pneumonia. In this report, the authors describe an unusual case of septic shock resulting from a combined *Legionella pneumophila* and *Streptococcus pneumoniae* infection associated with rhabdomyolysis, acute kidney injury, acute hypoxemic respiratory failure, pancreatitis, and acute liver injury.

## Introduction

Although polymicrobial etiology in patients with community-acquired pneumonia (CAP) is a growing clinical entity, the majority of bacterial CAP cases are caused by a single pathogen. Despite its infrequency, co-infections with *Legionella pneumophila* and *Streptococcus pneumoniae* have been previously reported in patients with CAP [1,2]. The role of co-infection and its influence on rates of complications and mortality is still unclear, albeit co-infections in patients with CAP may be a risk factor for intensive care unit (ICU) admission, severity of disease, and mortality. Legionnaires' disease is a life-threatening, common form of severe atypical pneumonia, with a mortality rate ranging from 5%-25% [3]. Several risk factors have been identified in patients with Legionnaires' disease, including male gender, extremes of age, smoking and chronic obstructive pulmonary disease (COPD), chronic alcohol consumption, acquired immunodeficiency syndrome (AIDS), hematologic and solid organ malignancies, diabetes mellitus, corticosteroids therapy, and end-stage renal disease (ESRD) [4,5]. Patients with Legionnaires' disease usually display a wide variety of multisystemic findings: central nervous system (CNS) abnormalities (headache, confusion, encephalopathy, lethargy), cardiac irregularities (relative bradycardia), gastrointestinal manifestations (watery diarrhea, abdominal pain), hepatic involvement (transient elevations of serum transaminases), renal abnormalities (microscopic hematuria, elevated creatinine), muscle involvement (elevated creatine phosphokinase), and electrolyte imbalance (hyponatremia, hypophosphatemia) [6]. Factors associated with increased mortality in patients with *Legionella* pneumonia are nosocomial acquisition, baseline immunosuppression (COPD, ESRD, AIDS, solid organ transplantation, diabetes mellitus, malignancies), delayed diagnosis and initiation of appropriate antimicrobial therapy, duration of symptoms before ICU admission longer than five days, severe hypoxia with needs for mechanical ventilation, and hyponatremia [5,7,8]. In this report, the authors describe an interesting case of a combined *Legionella pneumophila* and *Streptococcus pneumoniae* infection presenting with an extensive diversity of extra-pulmonary manifestations.

## Case presentation

**Figure 1:**
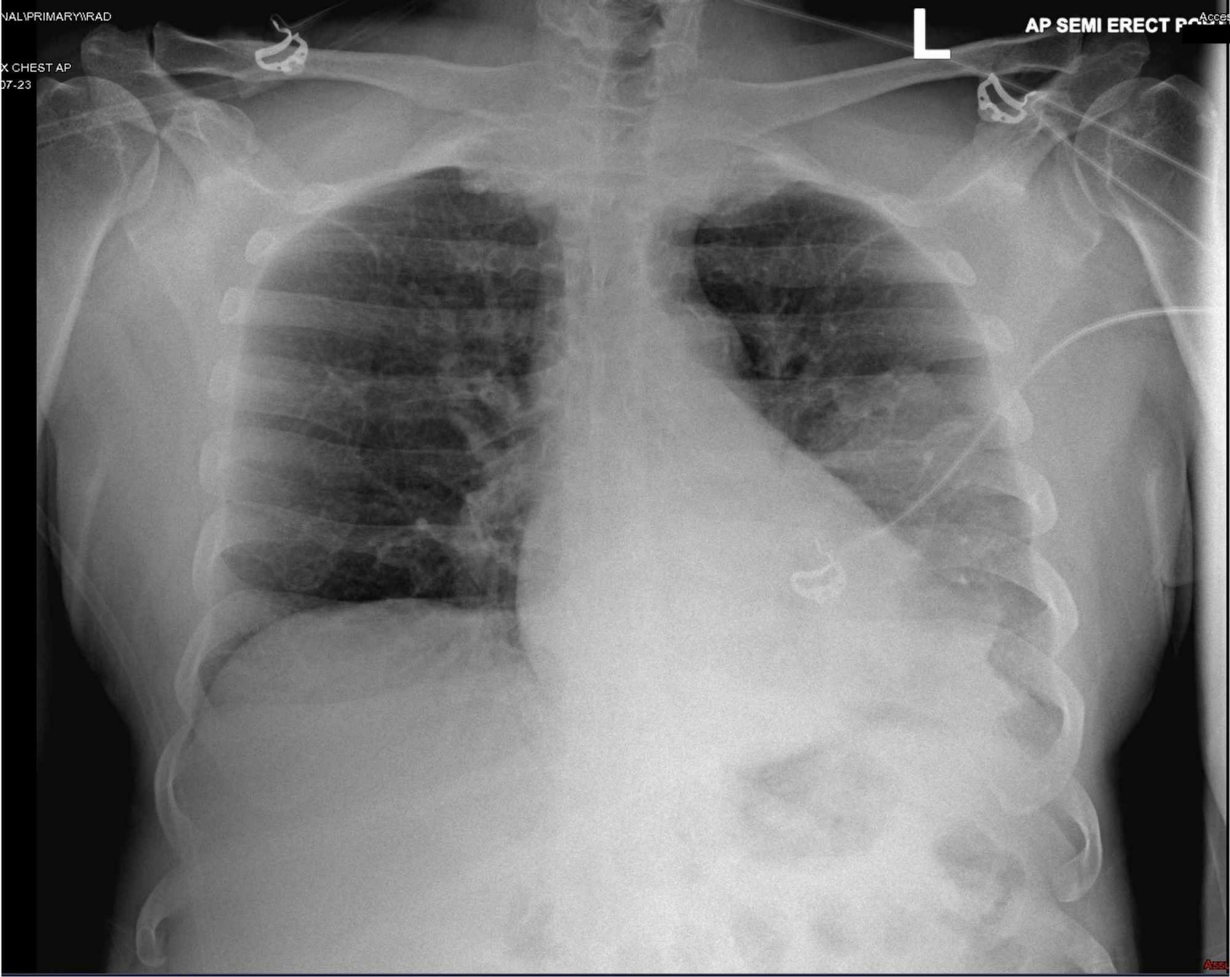
Chest X-ray demonstrating left lung opacity

**Figure 2:**
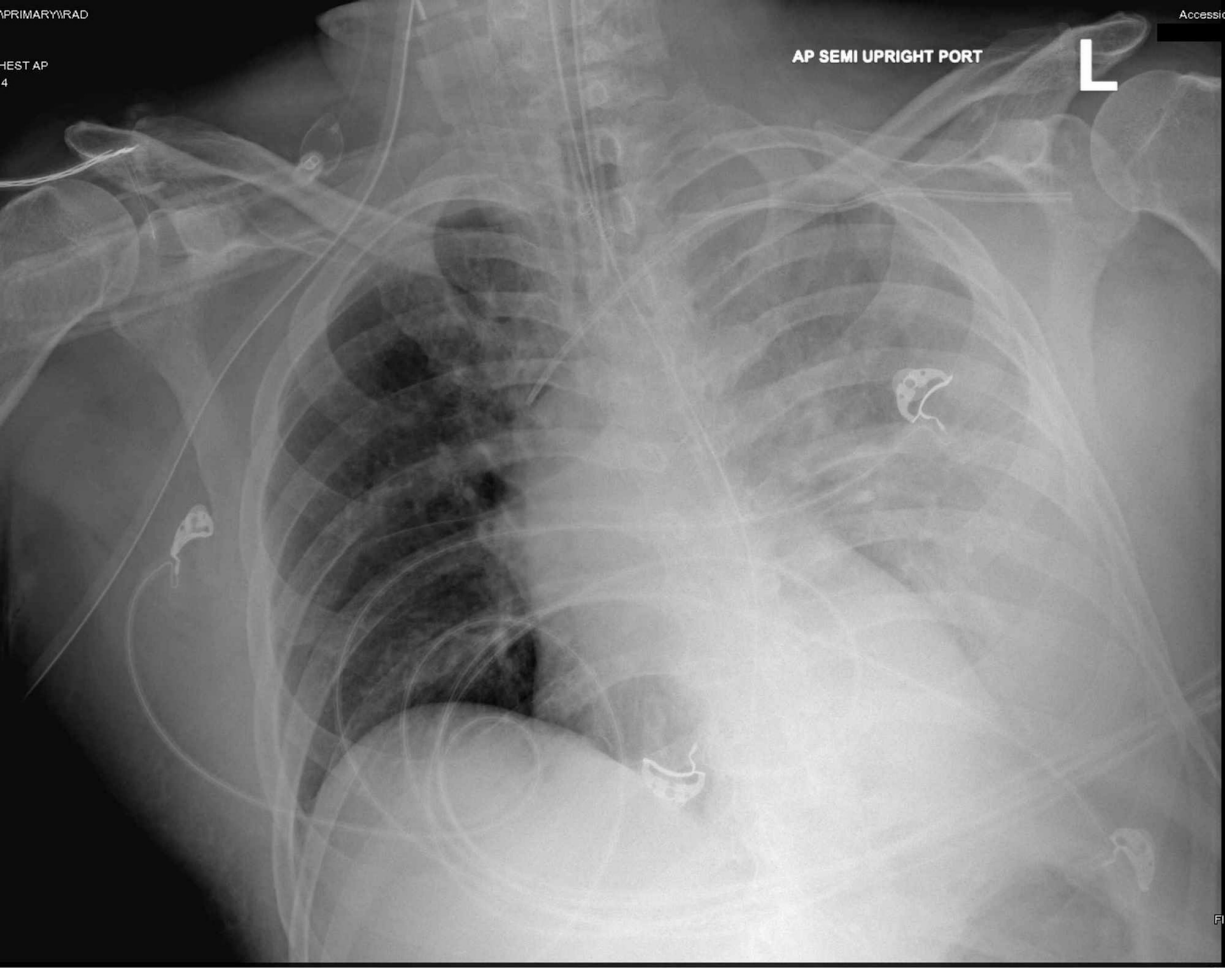
Chest X-ray showing worsening left lung opacity

A 65-year-old Hispanic male was brought to the emergency department (ED) complaining of worsening dyspnea, generalized malaise and weakness, and decreased oral intake for two weeks. His past medical history included systemic arterial hypertension, dyslipidemia, and COPD resulting from long-standing nicotine use. Previous pulmonary function test (PFT) results were not available, and the patient denied the use of supplemental oxygen therapy at home. The patient was alert, aware, and oriented to time, person, and place. He denied any recent travels or exposure to sick contacts. His vital signs on arrival to ED were as follows: blood pressure of 85/51 mmHg (mean arterial pressure of 58 mmHg), a respiratory rate of 28 breaths/minute, a heart rate of 114 beats/minute, a temperature of 102.8°F, and an oxygen saturation by pulse oximetry of 88% while breathing ambient air. Physical examination revealed decreased left lung air entry with rales on auscultation, and abdominal examination was unremarkable. Initial laboratory findings showed a white blood cell (WBC) count of 19,500 cells/mm^3^ (normal: 4,000-11,000 cells/mm^3^), a sodium level of 129 mEq/L (normal: 136-145 mEq/L), a bicarbonate level of 11 mEq/L (normal: 21-31 mEq/L), a blood urea nitrogen (BUN) level of 78 mg/dL (normal: 8-27 mg/dl), a serum ionized calcium level of 0.90 mg/dL (normal: 1.13-1.33 mg/dl), and a creatinine level of 7.7 mg/dL (normal: 0.8-1.3 mg/dl). Other abnormal laboratory results included an alanine aminotransferase (ALT) level of 80 U/L (normal: 7-52 U/L), a lipase level of 473 U/L (normal: 11-82 U/L), an alkaline phosphatase (AP) level of 173 U/L (normal: 34-104 U/L), a troponin level of 113 pg/mL (normal: 0-20 pg/ml), and a creatine phosphokinase (CPK) level of 1,103 U/L (normal: 38-174 U/L). Urine analysis showed large red-blood cells and proteinuria. Arterial blood gas (ABG) showed a pH of 7.22 (7.35-7.45), a pCO2 level of 25 mmHg (normal: 35-45 mmHg), and a paO2 level of 79 mmHg (normal: 75-100 mmHg), while patient receiving oxygen therapy through high-flow nasal canula at 60% fraction of inspired oxygen (FIO2). Lactic acid levels, lipid profile, total bilirubin, and coagulation studies were within normal limits, and urine toxicology screen was negative for recreational substances. Initial chest radiography (CXR) showed left lung opacity with consolidation (Figures 1, 2). A critical care medicine evaluation was requested, and the patient was admitted to ICU with the diagnosis of severe sepsis with septic shock likely resulting from CAP. Aggressive intravenous fluids resuscitation and vasopressors (norepinephrine, vasopressin) were initiated on arrival to ICU, and the patient was subsequently intubated and placed on mechanical ventilation because of worsening tachypnea and increased FIO2 requirements. Empiric intravenous antimicrobial therapy with ceftriaxone (2 g daily) and azithromycin (500 mg daily) was initiated, along with steroids (hydrocortisone 50 mg intravenously every six hours) and intravenous sodium bicarbonate (NaHCO3) infusion.

Electrocardiogram (ECG) showed sinus tachycardia without ST-segment or T-wave abnormalities. Transthoracic echocardiogram (TTE) demonstrated a normal left ventricular ejection fraction (LVEF), with a normal right ventricular systolic function. Abdominal ultrasound (US) showed a normal gallbladder with a normal common bile duct. Abdominal computed tomography (CT) with oral contrast revealed mild peripancreatic fat stranding suggestive of non-complicated pancreatitis and duodenitis and confirmed the presence of a left lung base consolidation.

Blood culture and respiratory culture were negative. Severe acute respiratory syndrome-associated coronavirus (SARS-CoV-2) test by polymerase chain reaction (PCR) was negative. *Legionella* as well as *Streptococcus pneumoniae* urine antigens, by immunochromatographic assay, were reported to be positive.

On day three of ICU admission, vasopressors were titrated off, metabolic acidosis had resolved, and the patient's creatinine,liver chemistries, and CPK levels were trending downward. The patient's FIO2 requirements were decreased to 45%, and he was extubated on day five of ICU admission. After 48 hours, he was transferred to a medical ward. The patient** **completed a total of 10 days of intravenous antimicrobial therapy with azithromycin 500 mg daily.

## Discussion

In this report, the authors described an uncommon case of a dual *Legionella pneumophila* and *Streptococcus pneumoniae* CAP, resulting in septic shock with multiorgan dysfunction syndrome and a few interesting extra-pulmonary manifestations. Based on a MEDLINE, PubMed search, and using the words “*Legionella*,” “*Streptococcus pneumoniae*,” “infection,” and “co-infection,” the authors identified a total of 30 published cases of combined *Legionella pneumophila* and *Streptococcus pneumoniae* co-infection in patients with CAP. Furthermore, our patient appears to be only the third case where a CAP co-infection was diagnosed based on positive urine antigens [2,9]. In this case, *Legionella pneumophila* infection may have preceded the *Streptococcus pneumoniae* infection or vice-versa; they may have been sequential infections or concurrent infections.

Besides presenting with acute hypoxemic respiratory failure requiring mechanical ventilation, a common occurrence in patients with severe CAP, the patient discussed in this article displayed a few compelling non-pulmonary findings. The patient’s elevated lipase levels were initially attributed to a decreased creatinine clearance in the setting of acute kidney injury (AKI) since initial symptoms did not include nausea, vomiting, or abdominal pain suggestive of pancreatic disease. Additionally, there was no history of alcohol consumption, no history of pancreatitis, lipid profile was within normal limits, and the abdominal US was unremarkable. Glomerular filtration is primarily responsible for the removal of lipase from serum. Decreased glomerular filtration and enlarged lipase molecule may artificially elevate serum lipase levels. However, abdominal CT with oral contrast showed evidence of acute pancreatitis. Interestingly, extensive literature research revealed previous reports of *Legionella* as the etiologic organism in acute pancreatitis [10-12]. On the contrary, *Streptococcus pneumoniae* has been reported as the causative pathogen responsible for infectious complications in patients with chronic pancreatitis (abscess, pseudocyst), but not as the primary causal microorganism in acute pancreatitis [13]. Although it is difficult to establish causality in this case, we postulate that *Legionella* might have been the possible etiology of pancreatitis in our patient.

Increased CPK levels were also observed in our patient on admission to ICU, at a range of 6.3 times above the normal upper limit. The authors hypothesized that elevated CPK levels might have been multifactorial in etiology: hypoxemia, hyperthermia, decreased perfusion to muscle tissue secondary to shock, and sepsis with possible direct invasion to muscles by *Legionella pneumophila* and *Streptococcus pneumoniae*. In a large study of 594 patients with CAP, the incidence of rhabdomyolysis was reported to be 2.4% (25 patients), with *Legionella pneumophila* (11 patients, 44%) and *Streptococcus pneumoniae* (four patients, 16%) accounting for the first and third most common etiologic microorganisms associated with rhabdomyolysis [14]. The etiology of rhabdomyolysis in patients with Legionnaires’ disease is unknown, but it may be related to direct muscle invasion by the microorganism and/or release of endotoxin into the bloodstream that may have a vasoconstrictive effect on small vessels leading to muscle injury. To the best of our knowledge, and after a substantial MEDLINE search, there have been 47 cases of *Legionella* spp. and 21 cases of *Streptococcus pneumoniae* infections reported in association with rhabdomyolysis. Excluding ours, that search yielded only two cases of rhabdomyolysis resulting from a combined *Legionella pneumophila* and *Streptococcus pneumoniae* infection.

Perhaps the most relevant finding in our patient was the critical degree of AKI and metabolic acidosis on admission to ICU. Fortunately, the patient responded adequately to aggressive intravenous fluid administration and intravenous NaHCO3 therapy without the need for renal replacement therapy with hemodialysis. The frequency of AKI in patients with Legionnaires’ disease ranges from 13% to 15% [15], with a reported mortality rate of more than 50% [16]. The mechanism of AKI associated with *Legionella* infection is probably multifactorial: intravascular volume depletion, shock, rhabdomyolysis, endotoxemia, and direct microorganism toxicity. In a review of 45 patients with Legionnaires’ disease and AKI, more than 50% of subjects required hemodialysis [16].

Although it was not considered critically important, our patient presented with hyponatremia on admission. In a comprehensive survey of CAP patients, Schuetz et al. reported sodium levels to be significantly lower in patients with Legionnaires’ disease as compared to patients with other CAP etiologies, with 44% of patients with Legionnaires’ disease displaying sodium levels <130 mEq/L [17]. The mechanism of hyponatremia in patients with Legionnaires’ disease is still unclear. Studies have shown a direct nephrotoxic effect of *Legionella* spp., in some cases leading to nephropathy with salt loss [18].

Mild elevation of troponins in our patient may have been related to a non-ST-segment elevation myocardial infarction (NSTEMI) and increased myocardial demands (type-2 myocardial infarction) resulting from hypoperfusion-shock and hypoxemia. Additional factors that may have contributed to elevated troponins, in this case, are rhabdomyolysis and AKI.

*Legionella* spp. are known to have hepatotropic activity. Autopsy studies have shown *Legionella* organisms within hepatic sinusoidal cells and reticuloendothelial system by electron microscopy [19]. We presumed that the acute liver injury in this case, as suggested by the elevation of liver enzymes, was likely multifactorial in etiology: sepsis, shock-hypoperfusion, and rhabdomyolysis. In a large cohort, more than 65% of patients with *Legionella* infection developed elevation of transaminases [5].

From the authors’ perspectives, the patient discussed in this manuscript is interesting because it highlights the distinctive, infrequently reported extra-pulmonary findings of an atypical co-infection with *Legionella pneumophila* and *Streptococcus pneumoniae*, which included acute pancreatitis, AKI, rhabdomyolysis, hepatocellular injury, and myocardial injury.

## Conclusions

In patients hospitalized with CAP, it may be pertinent to consider the possibility of polymicrobial infection. In patients with severe CAP and extra-pulmonary features, *Legionella* spp. should be considered as etiologic organisms. Therefore, *Legionella* and *Streptococcus pneumoniae* urine antigen tests should be requested on admission, and empiric intravenous antimicrobial therapy targeting those organisms must be initiated.
